# Supplementary material for: Culicoides allergens expressed in insect cells induce sulphidoleukotriene release from peripheral blood leukocytes of horses affected with insect bite hypersensitivity
Source: Front Immunol. 2025 Jun 6;16:1597233. doi: 10.3389/fimmu.2025.1597233 (PMC12178880; doi:10.3389/fimmu.2025.1597233)
Supplement: Supplementary Table 1 — List of Culicoides antigens used for production of specific antibodies in mice. [file Table1.docx]

**Supplementary Material**

**Supplementary table 1:** List of *Culicoides* antigens used for production of specific antibodies in mice.

**Supplementary table 2:** Breed and origin of the horses used in the study.
